# A Method for Selectively Enriching Microbial DNA from Contaminating Vertebrate Host DNA

**DOI:** 10.1371/journal.pone.0076096

**Published:** 2013-10-28

**Authors:** George R. Feehery, Erbay Yigit, Samuel O. Oyola, Bradley W. Langhorst, Victor T. Schmidt, Fiona J. Stewart, Eileen T. Dimalanta, Linda A. Amaral-Zettler, Theodore Davis, Michael A. Quail, Sriharsa Pradhan

**Affiliations:** 1 New England Biolabs Inc., Ipswich, Massachusetts, United States of America; 2 Wellcome Trust Sanger Institute, Cambridge, United Kingdom; 3 The Josephine Bay Paul Center for Comparative Molecular Biology and Evolution, Marine Biological Laboratory, Woods Hole, Massachusetts, United States of America; 4 Department of Geological Sciences, Brown University, Providence, Rhode Island, United States of America; 5 Department of Ecology and Evolutionary Biology, Brown University, Providence, Rhode Island, United States of America; Baylor College of Medicine, United States of America

## Abstract

DNA samples derived from vertebrate skin, bodily cavities and body fluids contain both host and microbial DNA; the latter often present as a minor component. Consequently, DNA sequencing of a microbiome sample frequently yields reads originating from the microbe(s) of interest, but with a vast excess of host genome-derived reads. In this study, we used a methyl-CpG binding domain (MBD) to separate methylated host DNA from microbial DNA based on differences in CpG methylation density. MBD fused to the Fc region of a human antibody (MBD-Fc) binds strongly to protein A paramagnetic beads, forming an effective one-step enrichment complex that was used to remove human or fish host DNA from bacterial and protistan DNA for subsequent sequencing and analysis. We report enrichment of DNA samples from human saliva, human blood, a mock malaria-infected blood sample and a black molly fish. When reads were mapped to reference genomes, sequence reads aligning to host genomes decreased 50-fold, while bacterial and *Plasmodium* DNA sequences reads increased 8–11.5-fold. The Shannon-Wiener diversity index was calculated for 149 bacterial species in saliva before and after enrichment. Unenriched saliva had an index of 4.72, while the enriched sample had an index of 4.80. The similarity of these indices demonstrates that bacterial species diversity and relative phylotype abundance remain conserved in enriched samples. Enrichment using the MBD-Fc method holds promise for targeted microbiome sequence analysis across a broad range of sample types.

## Introduction

From birth, humans participate in an intimate life-long relationship with their microbiome. Indeed, the number of microorganisms living in a human body is about 10-fold greater than the number of human cells [1]. Human-associated microbial communities affect diverse processes including digestion, immune system maturation, polysaccharide production, toxin degradation and pathogen defense [2]. Not all results of human-microbial interactions are positive; microbiota have been implicated as contributors to metabolic diseases through the modulation of host metabolism and inflammation. For example, bacteria have been implicated as a causative agent of atherosclerosis, which is associated with lipid accumulation and inflammation in the arterial wall [3]. Similarly, bacterial species are responsible for the two most common oral diseases in humans: dental caries (tooth decay) and periodontal (gum) disease [4]. Therefore, identification and characterization of complex microbial communities associated with humans is of increasing interest to the research community, medicine and public health.

It is now possible to study human-microbe relationships via DNA sequencing and analysis to establish identities, abundances, and functional characteristics of microbial community members [5], [6], but these studies are hindered by the complex nature of typical samples. Libraries prepared from many biological samples represent DNA from a mixture of bacteria, fungi (mainly yeasts), viruses, protists and an overwhelming amount of host genomic DNA. Nucleic-acid based techniques such as polymerase chain reaction (PCR), quantitative PCR (qPCR) and massively parallel sequencing offer rapid and highly sensitive options for detecting microbial species in collected specimens. As many microorganisms are difficult to grow or are unculturable [7], nucleic acid techniques offer significant advantages in breadth and depth of coverage. Currently, 16S ribosomal RNA gene-based sequencing can detect both abundant and rare members of a microbial community [8]; however, 16S rRNA gene approaches are not fully adequate for epidemiological studies or virulence factor identification. To circumvent the limitations of gene-based amplicon (e.g. 16S rRNA gene) sequencing, whole genome shotgun sequencing (WGS) has emerged as an alternative strategy for assessing microbial diversity [9]. One limitation of species identification by this method is the presence of large amounts of host genomic DNA in addition to microbial DNA. Metagenomics of clinical samples by direct sequencing or PCR can be inefficient and time consuming since most reads are derived from the host. Of particular relevance are clinical samples containing the malaria parasite, *Plasmodium falciparum*, which often contain greater than 90% human DNA.

In this report we describe a method for the separation of large pieces of DNA containing methyl-CpG from a complex mixture of human and bacterial DNA using the constant region of human IgG genetically fused to a human methyl-CpG binding domain (MBD-Fc) known to interact specifically with methyl-CpG elements [10], [11]. We demonstrate effective separation of vertebrate DNA from microbial DNA on the basis of differences in abundance of CpG methylation. We have analyzed the microbiomes of fish, human saliva, and human blood, as well as synthetic mixtures containing *Escherichia coli* and *Plasmodium falciparum* DNA with this method, and show evidence of relatively unbiased enrichment in all tested samples.

## Results

### MBD-Fc Fusion Selectively Depletes Human DNA from Mixed DNA Samples

Our MBD-Fc approach uses the following strategy: The methyl-CpG binding domain of human MBD2 protein was genetically fused to the Fc tail of human IgG1 (MBD-Fc). A truncated form of recombinant Protein A was covalently coupled to a paramagnetic bead and was used to bind the MBD-Fc protein. This complex selectively binds double-stranded DNA containing 5-methyl CpG dinucleotides [12].

**Figure 1:**
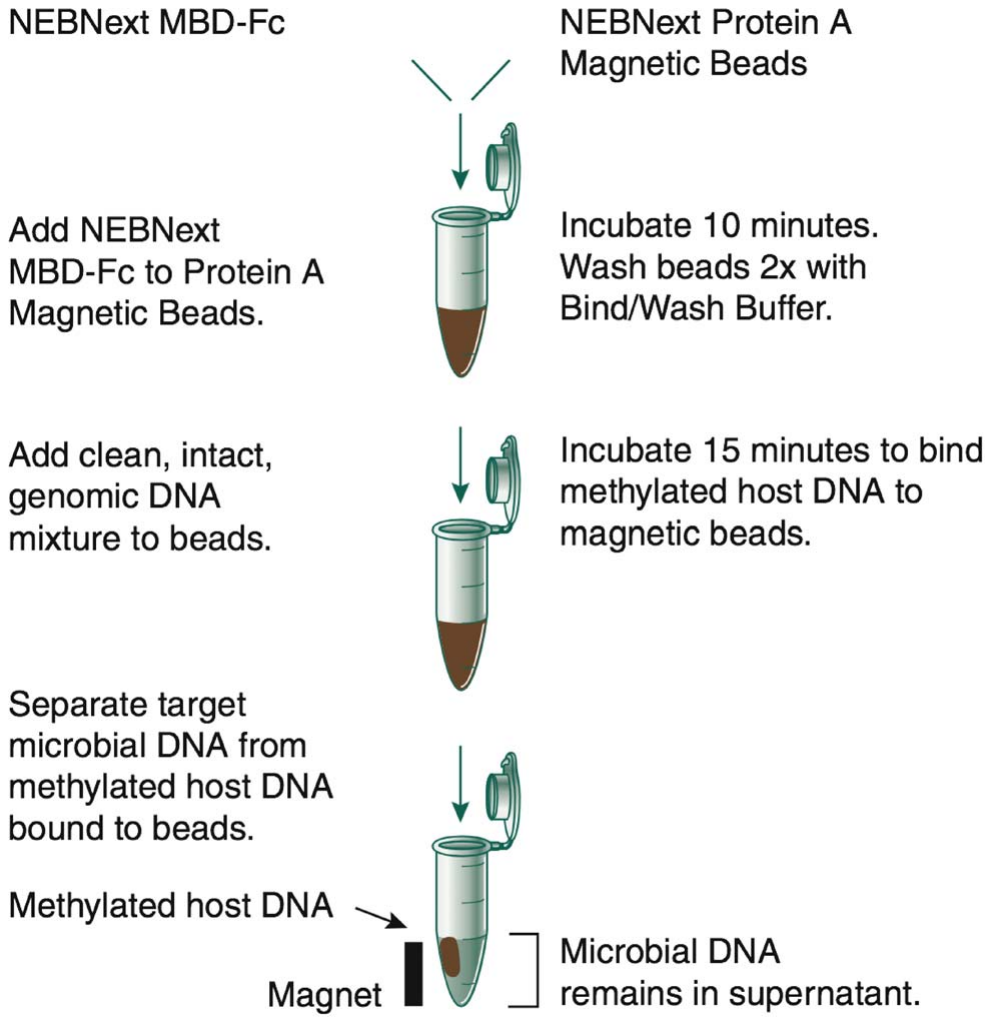
A schematic diagram of microbiome DNA enrichment using MBD-Fc.

**Figure 2:**
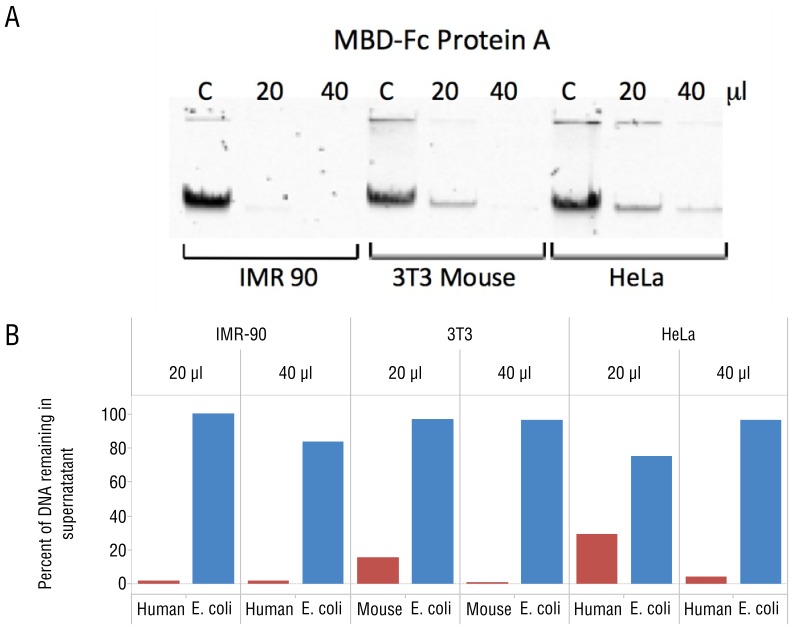
MBD-Fc fusions bind mammalian DNA. (A) Gel image demonstrating depletions of mammalian DNA by MBD-Fc binding. A total amount of 250 ng input DNA was incubated with increasing amounts of MBD-Fc beads as indicated above the gel. “C” corresponds to a control reaction with no MBD-Fc beads in the incubation mixture. The unbound, supernatant fraction of each mixture was resolved and is shown on the gel image. (B) Separation and quantitation of bound (mammalian) and unbound (*E. coli*) DNA in a mixture containing DNA from both sources. Gel densitometry results are shown with increasing bead quantity in the depletion experiment. The percentage of *E. coli* DNA in the supernatant was calculated by ^3^H scintillation counting and comparing with the input counts per minute.

To demonstrate specific interaction between the MBD-Fc fusion and methylated DNA, we prepared defined mixtures of ^3^H labeled *E. coli* K12 MG1655 DNA with mammalian genomic DNA from IMR-90, HeLa, and Mouse NIH 3T3 cell lines. These cell lines were chosen because their genomic DNA exhibit varying levels of CpG methylation density with IMR-90 being most dense, followed by NIH 3T3, and HeLa DNA being least dense [13]. Each mixture contained 10% ^3^H labeled *E. coli* DNA and 90% mammalian DNA (weight:weight). We first prebound the MBD-Fc protein with paramagnetic Protein A beads, then incubated increasing amounts of MBD-Fc bound Protein A with 500 ng of input DNA, and separated bead-bound from unbound DNA fractions using a magnetic field as outlined in Figure 1. Subsequently, we analyzed the amount of *E. coli* DNA in bound and unbound fractions with a scintillation counter, and mammalian DNA by gel densitometry measurements. We observed that mammalian DNA was efficiently depleted from the supernatant fraction after MBD-Fc enrichment. For the enrichment experiment in which we used 40 µL MBD protein, 1–4% of mammalian DNA remained in the supernatant for the IMR-90+*E. coli*, NIH 3T3+*E. coli*, and HeLa+*E. coli* mixed samples. Conversely, 84–100% of the ^3^H labeled *E. coli* DNA (lacking CpG methylation) remained in the supernatant fraction (Figure 2).

**Figure 3:**
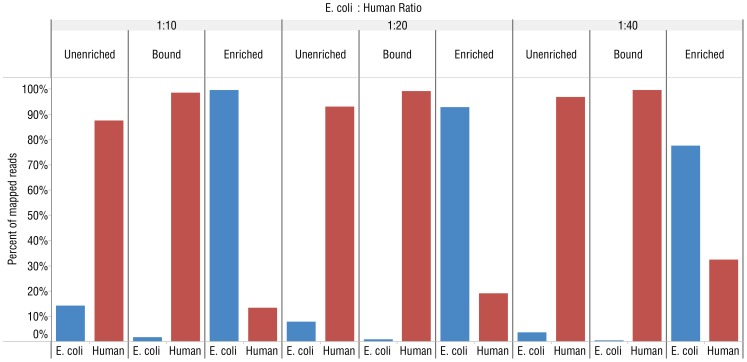
MBD-Fc enriches *E. coli* DNA from mixed *E. coli* and human DNA (IMR-90) samples. Graphs showing the percentage of mapped reads from Ion Torrent PGM experiments to either the *E. coli* MG1655 or human hg19 reference genome from libraries made with different ratios of human to *E. coli* DNA. The ratio between *E. coli* to human DNA in the premixed samples is indicated above the figure. “Unenriched” refers to untreated, control mixtures. “Bound” indicates DNA that remained bound to MBD-Fc beads and “Enriched” corresponds to unbound DNA remaining in the supernatant.

We further characterized and optimized the separation and enrichment of microbial DNA from human DNA using defined mixtures of human (IMR-90) and *E. coli* DNA with the MBD-Fc enrichment (Figure 1). 1–2 million reads from each sample were acquired on an Ion Torrent® PGM sequencer and aligned to a synthetic reference genome containing both the hg19 human reference sequence [14] and the *E. coli* MG1655 reference sequence [15] using Bowtie 2.0.4 [16]. We calculated the percentage of reads mapping to the *E. coli* genome vs. human chromosomes from an unenriched control sample, the enriched supernatant fraction and DNA eluted from the fraction bound to the magnetic beads. The starting unenriched DNA mixtures varied from 2.5–10% *E. coli* DNA. After the enrichment, 65–85% of the reads mapped to the *E. coli* reference genome with only a small percentage (15–35%) of reads mapping to the human reference (Figure 3). We also detected increased numbers of sequences mapping to the human mitochondrial genome in the supernatant fraction. While mitochondrial reads in the unenriched sample made up only 0.3% of human reads, mapped mitochondrial reads in the enriched sample made up 40% of human reads (Figure S1). As a further control, we eluted and sequenced DNA that remained bound to the beads after enrichment. Analysis of reads from these samples showed 97–99% aligned to human chromosomes, and 1–3% aligned to the *E. coli* genome. The small number of *E. coli* reads from the bead-bound samples was evenly distributed across the genome (data not shown).

**Figure 4:**
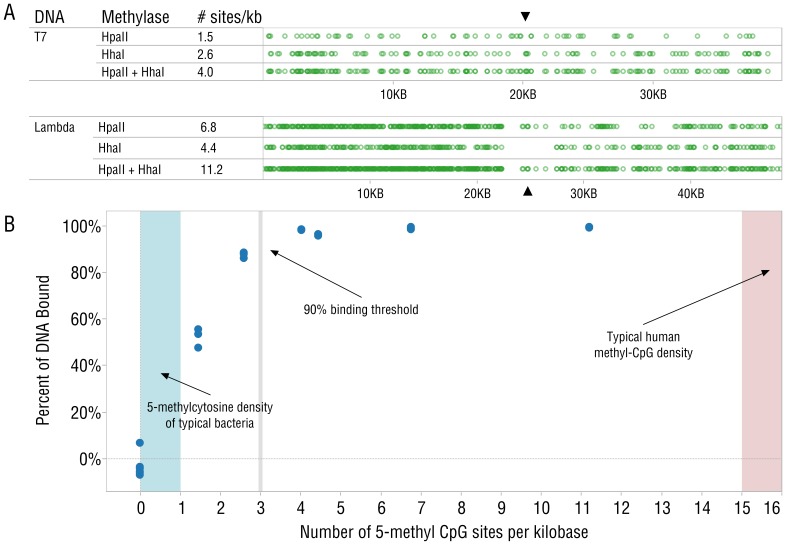
MBD-Fc beads bind DNA efficiently when methyl-CpG density of 20 kb fragments is greater than 3 methyl-CpG groups per kilobase. (A) Positions of CpG sites on T7 and Lambda DNA fragments (restriction sites indicated with ▴). (B) Graph representing the relationship between CpG site density and percent of DNA binding to the beads. Typical human methyl-CpG methylation density [17] (red) and total 5-methylcytosine levels found in *E. coli* (blue) are shaded and indicated with arrows. Since it is not reported to have CpG methylation, *E. coli* DNA is not expected to bind the beads. Boundaries for the bacterial reference area are derived from chromatography results of nuclease digested DNA [46], since this represents the maximum possible amount of CpG methylation. Replicates of each methylation density are overlaid in this plot.

To determine the CpG methylation density required for effective binding of DNA by the MBD-Fc-protein A complex, T7 (39.9 kb) and lambda phage (48.5 kb) DNAs were cleaved with BstEII and XbaI restriction enzymes, respectively. BstEII cuts T7 once, forming two ∼20 kb fragments; XbaI cuts lambda once, forming two ∼24 kb fragments. The T7 and lambda DNA fragments were then methylated with M.HhaI (GmCGC), M.Hpa II (CmCGG) or both methyltransferases. The resulting pool of six DNA fragments had methylation densities ranging from 1.5 to 14 methyl-CpG residues per kilobase (Figure 4A). From each pool, 250 ng of DNA was added to 40 µL of MBD-Fc-protein A beads, and the supernatant containing the unbound DNA was quantified using polyacrylamide gel electrophoresis and gel densitometry. Plotting the percentage of bound DNA vs. the number of methyl-CpG sites in these fragments reveals a threshold for efficient binding between 2 and 3 methyl-CpG per kilobase (Figure 4B). Lister *et al.* [17] report between 45 and 62 million methyl-CpG sites in IMR90 and H1 cell lines. Assuming that these cell lines reflect typical human methylation density and assuming even distribution, we can estimate ∼15–20 methyl-CpGs per kilobase of human DNA. Bacterial genomes generally do not contain sufficient CpG methylation density to efficiently bind MBD-Fc [18], [19].

### Human Microbiome Analysis of Enriched Samples from Saliva and Blood DNA

**Figure 5:**
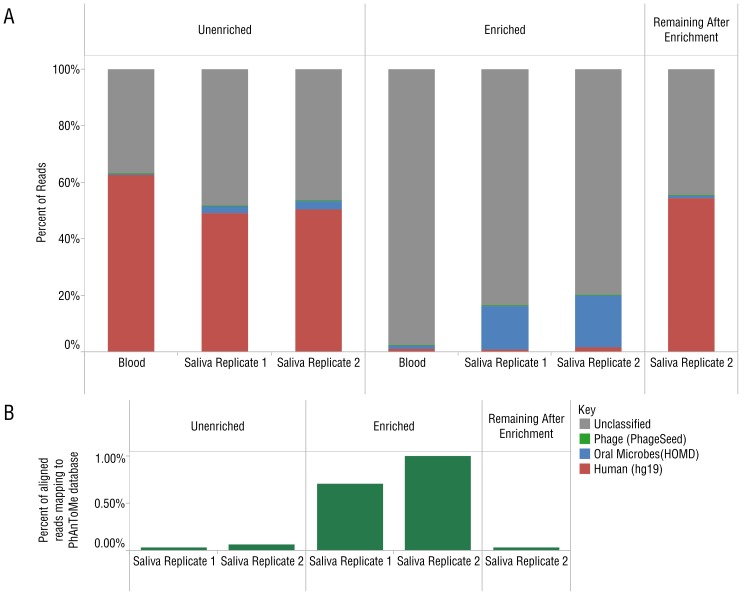
Analysis of SOLiD 4 sequence data from human saliva and blood samples before and after enrichment with MBD-Fc protein A paramagnetic beads. (A) Unenriched control, enriched and, remaining after enrichment (bead bound) samples are indicated above the figure. The data reflect reads mapping to known oral microbes in HOMD [20]. (B) Reads mapping to the PhageSeed database of viral sequences also show strong enrichment across two replicates.

**Figure 6:**
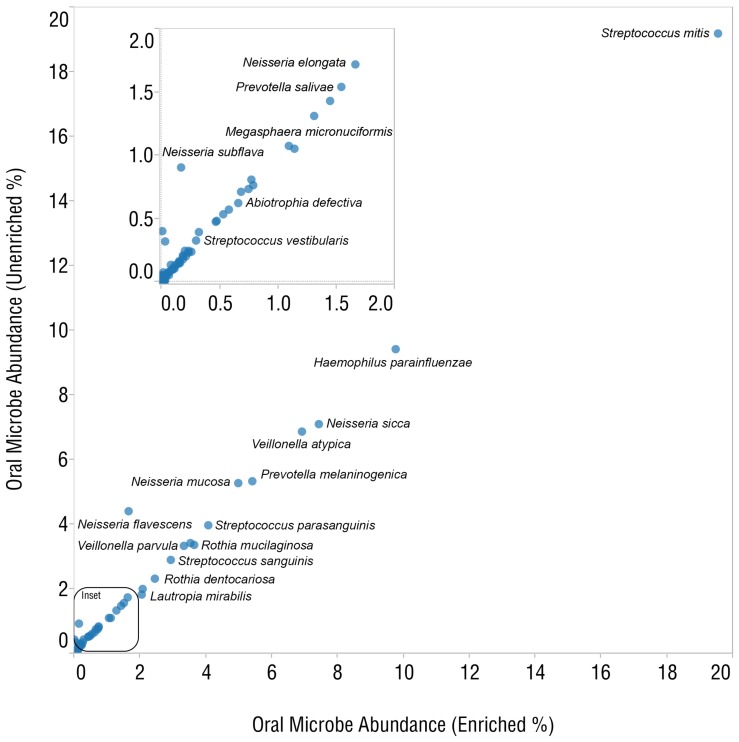
A concordance plot compares relative abundances of oral microbes between unenriched and enriched samples.

To determine the level of enrichment in biological samples, we analyzed microbiomes from DNA extracted from human blood and saliva samples before and after enrichment with the MBD-Fc protein bead complex. We produced libraries from enriched and unenriched samples, as well as from DNA that remained bound to the bead complex. All libraries were sequenced on the SOLiD® 4 platform, acquiring 174–346 million reads per blood sample and 501–537 million reads per saliva sample. Reads from the enriched library (unbound supernatant fraction) were compared with reads from the unenriched DNA library. Reads were aligned to the hg19 human reference genome [14], the Human Oral Microbiome Database (HOMD) [20], and the PhageSeed database of phage genomes (http://phantome.org). After enrichment, we observed a dramatic increase in reads mapping to the HOMD database (Figure 5A) and the PhageSeed database (Figure 5B). 94–96% of reads aligning to the human reference genome in the unenriched experiment were depleted after enrichment, corresponding to an 8-fold increase in reads mapping to the HOMD database. We also sequenced libraries prepared from the DNA that remained bound to the paramagnetic bead pellet. As expected, the vast majority of mapped reads from the bound fraction (99.3%) aligned to the human reference genome (Figure 5A). Plotting the abundance of known oral microbes observed by analysis with MetaPhlAn 1.7.1 [21] reveals high concordance between enriched and unenriched libraries for the most abundant species (Figure 6). Good concordance was maintained even as the limit of detection for low abundance population members was approached (Figure 6, inset). The predominant genera in saliva-extracted DNA were *Haemophilus, Streptococcus*, *Neisseria* and *Veillonella* (Table S1). In the commercial blood-derived DNA samples, *Pseudomonas*, *Escherichia* and *Acinetobactor* genera were predominant (Table S2). We also observed several common genera of bacteria in both saliva and blood microbiota including *Klebsiella*, *Haemophilus* and *Escherichia*. An analysis of phage sequences before and after enrichment in the saliva sample showed strong enrichment of many phage found in bacterial genera including *Streptococcus*, *Enterobacteria* and *Haemophilus* (Table S3).

### Microbial Species Diversity and Abundance is Similar by MBD-Fc Enrichment

**Figure 7:**
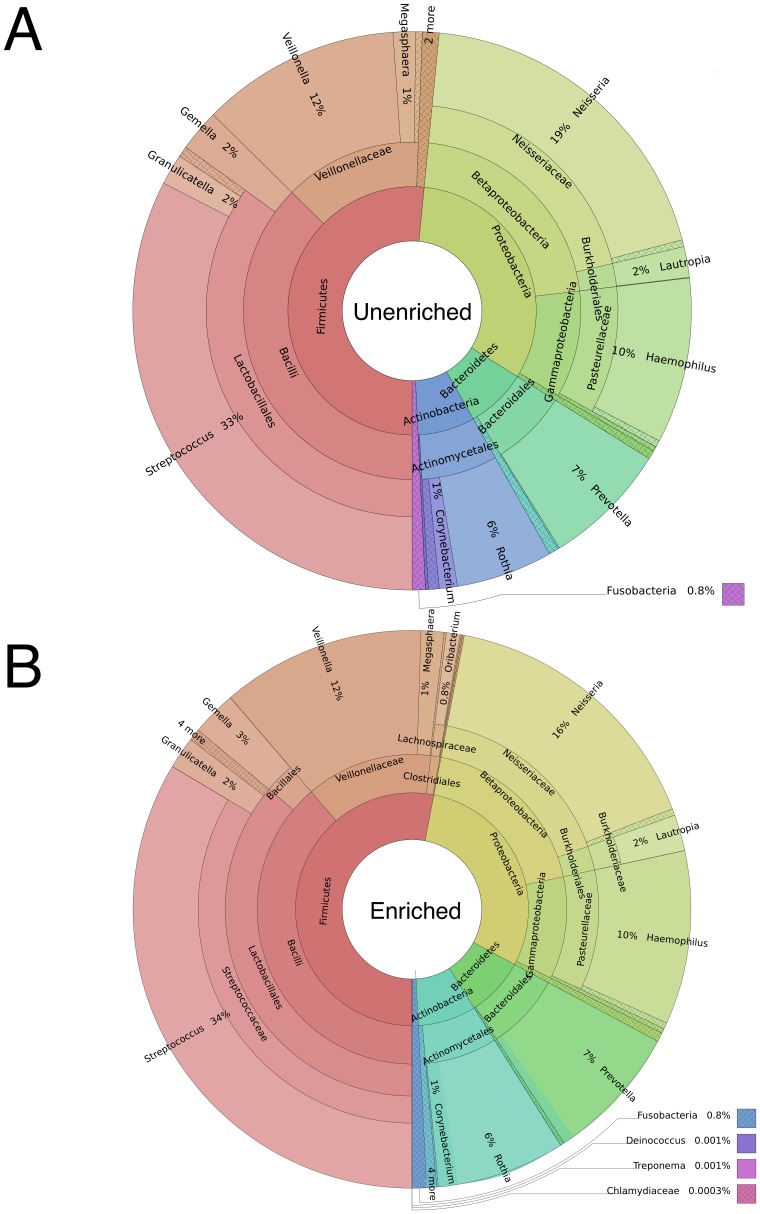
Hierarchical pie plots show that species and hierarchy abundances are maintained between (A) unenriched and (B) enriched samples.

The high abundance microorganisms from the saliva microbiome were very similar between the unenriched and MBD-Fc enriched samples. For example, the percentage of the total sequence reads matching microbial genomes in unenriched vs. enriched samples, respectively, were, 33% vs. 34% for *Streptococcus*, 19% vs. 16% for *Neisseria*, 12% vs. 12% for *Veillonella* and 10% vs. 10% for *Haemophilus* (Table S1). A Shannon-Wiener diversity index was calculated for 147 bacterial species in saliva that were observed in the unenriched (H′ = 4.72) and enriched (H′ = 4.80) datasets, indicating our enrichment method preserves diversity of the microbiome sample. We observed one abundant species, *Niesseria flavescens,* which was anomalous in the saliva enrichment. This organism may exhibit an unusual methylation density [22], allowing it to bind the enriching beads at a low level. Other *Niesseria* species (*N. mucosa, N. sicca* and *N. elognata*) are represented, but did not exhibit this anomalous enrichment. These data demonstrate relatively unbiased enrichment of microbial genomes after MBD-Fc enrichment. A strong correlation between the low-abundance microbial species in the unenriched and enriched samples is further evidence of unbiased enrichment. Additionally, we observed low abundance microbes that would have been difficult to detect without enrichment. *Deinococcus*, *Treponema* and *Bulleidia* sequence reads cumulatively represented less than 0.003% of the population after enrichment and were not detected by MetaPhlAn analysis in the unenriched samples (Figure 7A vs. 7B). Species reported at extremely low abundance (less than 0.01%) are derived from very few sequence reads and may represent false positives resulting from imperfect sequence data used to construct or query the MetaPhlAn database.

### Enrichment of *Plasmodium falciparum* DNA from Human-Plasmodium Mixture

**Figure 8:**
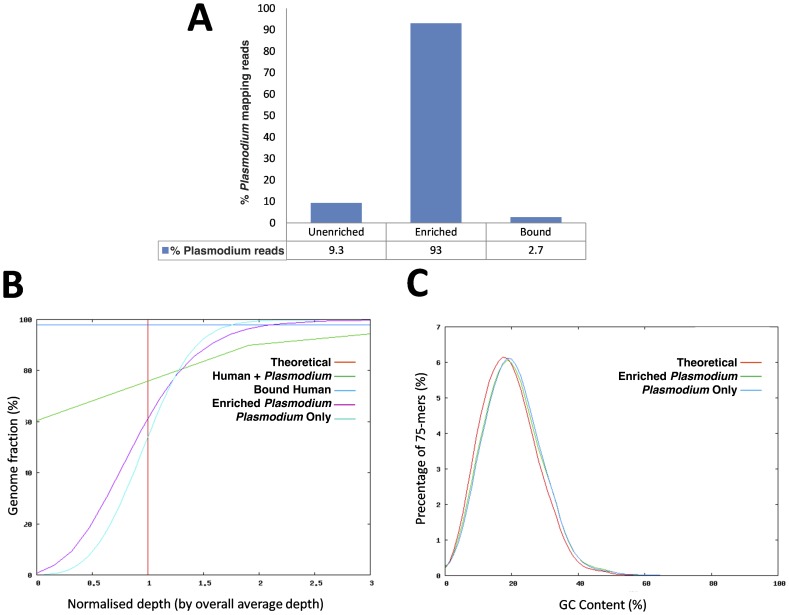
MBD-Fc separates human DNA from human-malarial DNA mixtures. (A) Graph of the percentage of 75 bp Illumina reads mapping to the *Plasmodium falciparum* reference sequence in the mixture before enrichment (Unenriched), after enrichment (Enriched) and the bound fraction following wash and elution as described above (Bound). (B) Evenness of coverage analysis metrics show enriched malaria reads (Enriched *Plasmodium*) in line with pure malaria DNA reads (*Plasmodium* Only). Unlike the unenriched input sample (Human + *Plasmodium*) showing 60% of the *Plasmodium* genome uncovered (zero depth), enriched sample (Enriched *Plasmodium*) showed even coverage of the genome with no regions lacking coverage. The amount of *Plasmodium* DNA retained in the pellet (Bound Human) following wash and elution was insignificant. (C) GC-content and bias analysis. No base bias was detected in the enriched sample. Average GC content of the enriched sample (Enriched *Plasmodium*) matches the pure malaria sample (*Plasmodium* Only) and is very close to the theoretical GC coverage (Theoretical).

MBD-Fc was effective in separating human host contamination from bacterial DNA based on differential CpG methylation density. Therefore, we hypothesized that *Plasmodium falciparum* DNA could also be enriched from human host genomic DNA. To test this, a mock sample (containing 90% human and 10% *P. falciparum* DNA) was used in our MBD-Fc enrichment workflow (Figure 1), and samples were sequenced on the Illumina HiSeq 2000 and MiSeq instruments. Analysis of reads from enriched and unenriched samples indicated an 8-fold increase in the number of reads from the enriched *Plasmodium* DNA (Figure 8A). Further analysis of the sequence reads obtained from the enriched sample showed no significant bias in either overall genome-wide coverage (Figure 8B) or GC content (Figure 8C).

### Black Molly Fish Microbiome can be Enriched using MBD-Fc Beads

**Figure 9:**
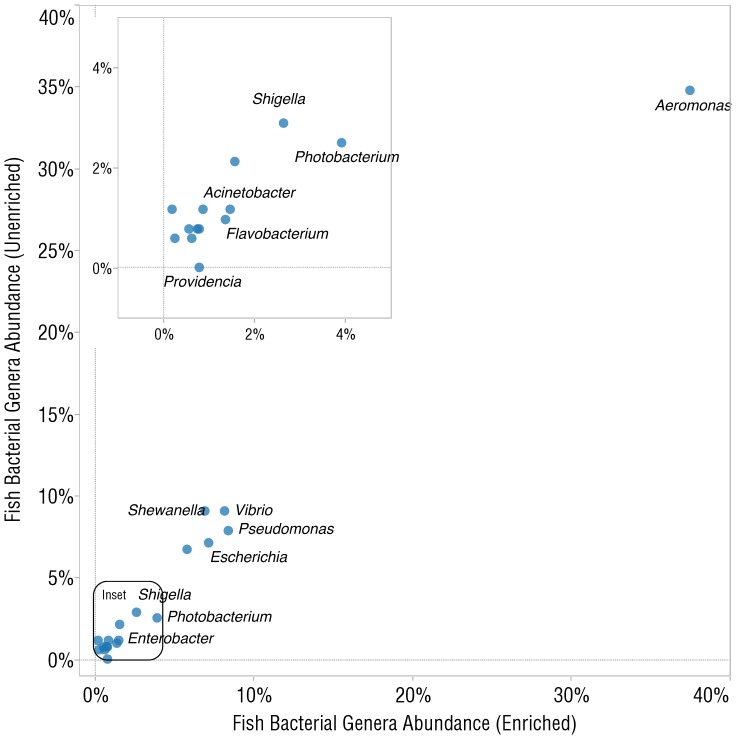
Analysis of Illumina sequence reads of a black molly (*Poecilia* cf. *sphenops*) whole fish DNA library before and after enrichment with MBD-Fc protein A paramagnetic beads. Shown is a concordance plot comparing relative abundances of microbial genera between the enriched and unenriched samples.

Since most vertebrate genomes contain methyl-CpG dinucleotide residues, we hypothesized that MBD-Fc based beads would also be effective at enriching for microbial DNA in other vertebrate samples. We enriched DNA derived from an entire black molly fish (*Poecilia* cf. *sphenops*), prepared libraries, and sequenced them on Illumina GAIIx and MiSeq sequencers. We analyzed the microbiome of the black molly using the MG-RAST [23] server and compared relative abundance of microbes between unenriched and enriched samples and observed an even enrichment, as demonstrated by the concordance plot (Figure 9). The most abundant genera of bacteria reported were *Aeromonas, Pseudomonas, Vibrio,* and *Shewanella*. Reads were also analyzed by MetaPhlAn, which reported the most abundant genera in the same order as MG-RAST (Table S4).

Enrichment of microbial DNA from black molly fish using MBD-Fc beads also gave us sufficient sequence coverage of microbial genomes to build meaningful assemblies and high quality protein annotations. Our 50-bp single end metagenomic reads were assembled using the CLC de novo assembler [24] and yielded fungal, viral and bacterial contigs greater than 4000 bp, often with upwards of 20 fold coverage. Analysis of protein predictions using the MG-RAST server [23] yielded 198 contigs with more than 10 fold coverage matching bacterial derived drug resistance genes. More than 15 drug resistant genes derived from each of *Aeromonas hydrophila*, *A. salmonicida*, *Vibrio cholerae* and *E. coli* were also detected. These species are all known fish pathogens, and are likely targeted by antibiotic treatment as regular practice in the ornamental fish industry [25]–[27]. The most abundant drug resistance related protein had 49× coverage over a 1,390 bp contig and was annotated as the quinolone resistance protein QnrS2, derived from another the fish pathogen *A. caviae*. Quinolones are powerful broad-spectrum antibiotics with known usage in aquaculture, and QnrS2 has been previously implicated in antibiotic resistance in ornamental fish [25].

## Discussion

We have described a robust, even, and broadly applicable enrichment method taking advantage of the properties of a methyl-binding domain genetically fused to the constant region of human IgG and linked to paramagnetic beads. This protein is known to specifically interact with 5-methyl CpG motifs found in vertebrate DNA but largely absent from microbial and mitochondrial DNA [28], [29]. DNA fragments having sufficient CpG methylation (greater than ∼3 sites/kilobase) efficiently bind MBD-Fc conjugated magnetic beads and can be quickly separated from less methylated DNA, which remains in the supernatant. We have demonstrated the utility of this method in a series of experiments of varied complexity. We began with experiments on DNA fragments of controlled methylation, providing evidence of the mechanism and capabilities of the MDB-Fc enrichment method. Next, mixtures of ^3^H-labeled *E. coli* DNA with mammalian DNA were examined and showed that this technique is effective in a variety of cell types. Sequencing and analysis of experiments on defined mixtures of human and microbe DNA characterized the specificity of this method. Finally, we report experiments on a variety of real samples including human blood, human saliva, and fish, showing unbiased and robust enrichment.

In addition to the method described in this paper, we are aware of two other strategies available for enriching microbial DNA from human samples, both of which have been available commercially. The MolYsis® (Molzym GmbH & Co. KG, Bremen, Germany) strategy takes advantage of differential lysis of human and microbial cells. In short, human cells are lysed under chaotropic conditions, human DNA is removed by enzymatic digestion, the enzyme is then removed from the sample, and finally microbial cells are lysed and microbial DNA is purified. The MolYsis method could introduce bias in selection of microbial DNA since some bacteria are likely to lyse under the same conditions as human host cells, changing the apparent microbial composition [30]. Another method, Pureprover® (previously available from SIRS-Lab GmbH, Jena, Germany), uses conventionally extracted microbiome/host DNA, and a protein to bind non-methylated CpG motifs in bacterial genomes [30]. Since microbes have a range of density of CpG motifs, capture efficiency of microbial DNA may vary, changing the microbiome species distribution in the analyzed sample.

Other methods of eliminating human DNA contamination from clinical samples (e.g. malarial patient samples) have various limitations [31]. Alternative methods might be specific to a single organism, involve a laborious and bias-prone culture step or employ complex lab techniques [32]. A method utilizing a methyl-cytosine dependent restriction enzyme (e.g. MspJI) to enrich *Plasmodium* DNA in malarial DNA samples was recently reported [33]. This approach uses a restriction enzyme requiring a methylated recognition sequence to selectively digest host DNA contamination in malarial samples. The method depletes 80% of the host DNA and enriches the *Plasmodium* DNA 9-fold but requires a 16 hour incubation at 37°C. Since malaria is often found in regions where sample collection, storage and processing is difficult, a more desirable approach would involve blood collection followed by DNA extraction of the clinical sample in the field for subsequent enrichment and analysis in a laboratory.

Many organisms present in vertebrate microbiomes are not culturable; therefore, high throughput sequencing of 16S ribosomal RNA gene amplicons is often used for characterization of natural communities. However, 16S ribosomal RNA gene sequencing only provides information about identity and abundance of community members without considering the substantial additional information available in the bacterial genomes. Additionally, 16S rRNA gene methods cannot be used to simultaneously detect both bacteria and the non-bacterial microbes containing 18S rRNA genes [34], [35]. Other culture independent techniques such as whole genome shotgun sequencing (WGS) are being used more frequently to identify organisms, provide insights into gene function and allow for inference of the functional potential of a microbial population. The presence of host DNA in such samples can result in a significant number of sequencing reads that must be discarded since they do not contain information about the microbial community. In the absence of an enrichment step, extensive sequence data must be obtained in order to achieve sufficient coverage of the sample of interest. In a recent report, analysis of the human salivary microbiome using WGS sequencing revealed a large fraction of the BLASTN [36] hits matched to human DNA, and only a small fraction represented bacterial and viral sequences amounting to 0.73% and 0.0036%, respectively [37]. Additionally, in 2012, the Human Microbiome Project Consortium [38] reported especially high levels of human DNA in soft tissue samples such as mid-vagina, anterior nares and throat; as well as high levels from DNA extracted from saliva. In our study of saliva-associated microbes, we observed low abundance of host sequences and high abundance of bacterial genera including *Rothia, Streptococcus, Haemophilus, Veillonella,* and *Neisseria*, among more than 100 total observed species.

We also observed strong enrichment of human mitochondrial DNA in both saliva and blood libraries. Mitochondrial and chloroplast DNA have been previously reported to have very low abundance of CpG methylation [28], [29], [39], [40]. Although recent literature suggests that some CpG methylation is present in mitochondrial DNA [41], [42], our observation of strong enrichment of mitochondrial DNA leads us to believe that the level of CpG methylation in the samples we have studied (blood, saliva, and cell lines) cannot be sufficient to promote efficient binding of the MBD-Fc beads. Similarly, we have observed strong enrichment of chloroplast DNA in plant samples using MBD-Fc based enrichment steps (data not shown). Furthermore, analysis of phage sequences before and after enrichment revealed increases in sequence reads from many species of phage with *Streptococcus*, *Enterobacteria* and *Haemophilus* phage being the major species.

Our studies with blood enrichment are complicated by the fact that the source material was from a commercial vendor with no specifications of collection date, source information (normal, disease or surgical patient) or method of storage prior to shipment. Bacteria from the genus *Pseudomonas* represented the majority of reads in the enriched dataset, which could reflect the presence of these organisms in the original host, but may represent environmental contamination during or after sample collection.

MBD-Fc bead enrichment is effective in non-human vertebrates as well. Various bacterial genera including *Aeromonas, Pseudomonas, Vibrio,* and *Shewanella* were observed in high abundance in our study of the black molly fish. We were not able to robustly assign reads to specific bacterial species, probably due to short read lengths and low representation of fish-associated bacteria in databases.

Depletion of methylated DNA using paramagnetic bead-coupled MBD-Fc is a simple and rapid procedure that can easily be automated or performed with minimal equipment. We have demonstrated that this method can be used to separate host DNA from microbial DNA in a variety of contexts (human saliva, human blood, black molly fish, and artificial mixtures of human and *E. coli*). In addition, we have successfully enriched *P. falciparum* DNA from a prepared mixture of human and *P. falciparum* DNA, demonstrating its practicality for improving the laborious enrichment of *Plasmodium* DNA in patient blood samples. We have observed efficient separation of low-methylation density DNA from DNA with higher 5-methyl CpG content in varied contexts. We expect the MBD-Fc bead method to be a robust method of separating microbial DNA from plant, animal, or any host organism DNA containing sufficient CpG methylation. Finally, through sequencing data analysis, we have shown that this enrichment method evenly enriches microbial DNA, accurately reflecting the diversity of microbial species in the original sample.

## Materials and Methods

### Ethics Statement

This study was carried out in strict accordance with the recommendations in the Guide for the Care and Use of Laboratory Animals of the National Institutes of Health. The black molly (*Poecilia* cf. *sphenops*) protocols were conducted under the Marine Biological Laboratory IACUC protocol #12–35 by Linda Amaral-Zettler. Fish were maintained under the supervision of an institutional veterinarian, and sick or moribund fish were euthanized immediately to minimize suffering. To minimize suffering at the point of sacrifice, fish were euthanized immediately upon collection using a 250 ppm buffered Tricaine methanesulfonate (MS-222) solution.

### Genomic DNA Preparation

*E. coli* K-12 MG1655 and IMR-90 cells (ATCC CCL-186) were suspended in 50 mL of lysis buffer, 50 mM Tris (pH 7.5), 20 mM EDTA, 1% SDS, 100 µg/mL Proteinase K (NEB #P8102S), and incubated at 50°C for 3 hours with frequent mixing. DNA was extracted from samples once with Tris-EDTA equilibrated phenol and once with methylene chloride [43]. DNA was precipitated with two volumes of ethanol and washed twice with 70% ethanol. The precipitates were then centrifuged at 14,000×g for 15 minutes at 4°C and the pellets were air dried at room temperature and suspended in 200 µL of 1× TE buffer (pH 7.5). Twenty µL of 0.4 mg/mL RNase A was added and the samples were incubated at 37°C for one hour. The samples were phenol/methylene chloride extracted, ethanol precipitated as above and DNA pellets were suspended in 1× TE buffer (pH 7.5). The final concentration of genomic DNA was adjusted to 100 µg/mL. Care was taken in pipetting steps to avoid unnecessary fragmentation of genomic DNA. DNA quality and quantity were assayed by agarose gel electrophoresis of the samples alongside a DNA marker (2-log DNA ladder, NEB #N3200S) and by Nanodrop® spectrophotometry.

### Preparation of ^3^H *E. coli* DNA

*E. coli* cells were grown in Luria broth (LB) tetracycline and tritiated thymidine (Moravek Biochemicals MT 6036) for 6–8 hours at 30°C in a shaker incubator (300 rpm). Then, bacterial cells were pelleted at 14,000×g for 10 min. in a centrifuge. DNA was extracted by phenol/chloroform and precipitated by ethanol. Specific activity of labeled *E. coli* DNA was adjusted to 30,000 cpm/µl by mixing with unlabeled *E. coli* DNA.

### Metagenomic DNA Preparation

25 mL of human buffy coats/leukocytes (Innovative Research) were suspended in 50 mL of lysis buffer and genomic DNA was prepared as described above. 500 mL of pooled human saliva (Innovative Research) was centrifuged at 14,000×g for 15 minutes at 4°C, the pellet was resuspended in 50 mL of lysis buffer, and genomic DNA was prepared as described above. Agarose gel analysis revealed ∼50% of DNA from the pooled saliva sample was shorter than 10 kb. To further purify the DNA and enrich for high molecular weight fragments, the sample was loaded on a 1% low melt agarose gel with 1× SYBR® Safe DNA Gel Stain (Life Technologies). The ∼15 kb band was cut out of the gel. The resulting gel slice was melted at 50°C and cooled to 42°C. 200 µL of 10× β Agarase I buffer and 20 units β Agarase (NEB #M0392S) were added to the sample and incubated at 42°C for 30 minutes. DNA was precipitated using 2 volumes of ethanol, air dried at room temperature and resuspended in 1× TE buffer (pH 7.5) to a concentration of 100 µg/mL.

### Black Molly DNA Preparation

A single ∼5 cm black molly was euthanized in MS-222, rinsed and homogenized in 30 mL 1× PBS with dissection scissors. The homogenate was vortexed for 10 minutes to disassociate microbial cells from host tissue, and then filtered through a 5-µm Isopore™ polycarbonate filter (Millipore part no. TMTP02500). The filtrate was centrifuged at 14,000×g for 10 minutes to pellet microbial components of the fish sample. The pellet was then dried and resuspended in 600 µL Gentra® Puregene Yeast/Bact. Kit cell lysis solution (Qiagen #158722), and DNA purification was performed according to the manufacturer’s protocol.

### Simulation of Clinical Malaria Samples

For the malaria DNA enrichment, *P. falciparum* 3D7 genomic DNA was obtained from Prof. Chris Newbold’s laboratory at the University of Oxford, UK. Human genomic DNA was purchased from (Promega #G3401). Mock samples were manually prepared by mixing 0.2 µg of *P. falciparum* 3D7 genomic DNA with 1.8 µg of human genomic DNA to obtain 2 µg of a simulated clinical genomic DNA sample.

### Prebinding of MBD-Fc Protein to Protein A Paramagnetic Beads

Protein A paramagnetic beads (NEB #E2612, NEB #E2615A) were uniformly suspended in bind/wash buffer (NEB #E2612, NEB #E2616A) by gentle pipetting. 1 mL of the suspension was transferred to a 1.5 mL microcentrifuge tube, and 100 µL of MBD-Fc protein solution (NEB #E2612, NEB #E2614A) was added into the tube. The paramagnetic beads and MBD-Fc protein mixture was gently rotated for 10 minutes at room temperature. The tube was placed in a magnetic separator at room temperature until the supernatant was clear and beads were collected on the wall of the tube (5 minutes). The supernatant was removed and discarded using a pipette without disturbing the beads. 1 mL of 1× ice-cold bind/wash buffer was added to the tube to wash the beads, the tubes were removed from the rack, and the solution was pipetted up and down three times. The sample was mixed on a rotating mixer for 3 minutes at room temperature, and then briefly centrifuged. The tube was placed in a magnetic separator at room temperature until the supernatant was clear and beads were collected on the wall of the tube (5 minutes). The supernatant was removed and discarded using a pipette without disturbing the beads. The wash step was repeated. After the final wash, beads were resuspended in 1 mL of ice cold 1× wash/bind buffer and kept at 4°C for no more than 7 days.

### Enrichment of Microbial DNA

The purified sample DNA prepared as described above was mixed with MBD-Fc protein A beads in a ratio of 1 µg of sample DNA to 160 µL of beads. The sample DNA was directly added to the bead slurry and incubated for 15 minutes at room temperature with gentle rotation. The incubated mixture was placed on a magnetic rack at room temperature until the supernatant was clear and beads were collected on the wall of the tube (2–5 minutes). The supernatant, containing enriched microbial DNA was carefully removed with a pipette without disturbing the beads, purified by 1.8× volume of Agencourt AMPure® XP beads (Beckman Coulter #A63880) according to the manufacturer’s instructions and the DNA was eluted in 150 µL 1× TE buffer (pH 7.5). Volumes for this procedure were scaled directly depending on the amount of input DNA.

For the samples described in this study, the following specific input amounts used for the microbial DNA enrichment experiments were as follows: saliva microbiome enrichment, 12 µg of input DNA was enriched using 2 mL of MBD-Fc Protein A paramagnetic beads; black molly microbiome enrichment, 9.12 µg of input DNA was enriched using 1.5 mL of MBD-Fc Protein A paramagnetic beads; and for *Plasmodium falciparum* DNA enrichment, 2 µg Plasmodium falciparum/human DNA mixture was enriched using 320 µL of MBD-Fc Protein A paramagnetic beads.

### Elution of Host DNA

Host DNA bound to the MBD-Fc protein A beads during the enrichment of microbial DNA procedure was recovered for further analysis. After the supernatant-containing enriched microbial DNA was removed (described above), any remaining liquid was removed from the bottom of the tube without disturbing the beads and was discarded. The beads were rinsed with 1 mL 1× ice-cold wash/bind buffer, placed on a magnetic rack at room temperature until the supernatant was clear, beads were collected on the wall of the tube (2–5 minutes) and the supernatant was discarded. The beads were then resuspended in 150 µL 1× TE buffer (pH 7.5), and 15 µL of Proteinase K (NEB #P8102) was added before incubating at 70°C for 20 minutes with occasional mixing. The tube was briefly centrifuged and placed on a magnetic rack at room temperature until the supernatant was clear and beads were collected on the wall of the tube (2–5 minutes). The supernatant containing methylated host DNA was removed, purified by 1.8× volume of Agencourt AMPure® XP beads (Beckman Coulter #A63880) according to the manufacturer’s instructions and the host DNA was eluted in 100 µL of 1× TE buffer (pH 7.5).

### Preparation of Next-generation Sequencing Libraries

DNA extracted from saliva and leukocytes were used to generate SOLiD 4 libraries using NEBNext® Fast DNA Library Prep Set for Ion Torrent (NEB #E6270) with SOLiD 4 adaptors and primers; mixtures of DNA extracted form *E. coli* K-12 MG1655 and IMR-90 cells were used to generate Ion Torrent libraries using NEBNext Fast DNA Library Prep Set for Ion Torrent (NEB #E6270); DNA extracted from black molly was used to generate Illumina paired end sequencing libraries using NEBNext Ultra DNA Library Prep Kit and Multiplex Oligos for Illumina (NEB #E7370 and #E7335). Briefly, genomic DNA was sheared using a Covaris S2 device to obtain an average fragment size of ∼200 bp. Sheared DNA was end repaired, dA-tailed (Illumina libraries only), ligated to platform specific adaptors, size selected to an average size of ∼300–350 bp by Agencourt AMPure XP beads (Beckman Coulter #A63880), and amplified by 6 to 12 cycles of PCR.

Illumina libraries for the *Plasmodium falciparum* enrichment experiment were prepared as follows: Genomic DNA (500 ng) was sheared using a Covaris S2 device to obtain an average fragment size of ∼350 bp. Illumina paired-end sequencing libraries were constructed using the NEBNext DNA Library Prep Reagent Set for Illumina (NEB #E6000) following the standard Illumina sample preparation protocol. PCR library amplifications were performed with an MJ Research Thermo Cycler PTC-225. Illumina PE 1.0 and 2.0 primers or PE 1.0 and 2.0-derived indexing primers were used to amplify adapter-ligated library fragments by PCR. Libraries were amplified using optimized PCR conditions described in [44].

### Calculation of Percent Read Metrics

Unless otherwise specified, percent enrichment was calculated as below using only mapped reads.

Percent microbe reads = (number of microbe reads/(number of microbe reads + number of host reads)) * 100.

Percent host reads = (number of host reads/(number of microbe reads + number of host reads)) * 100.

### Ion Torrent Sequencing of Defined Mixtures

Fastq files were processed to remove reads shorter than 50 base pairs and mapped to a combined reference sequence database containing both the hg19 human reference genome and the *E. coli* MG1655 genome. Reads were mapped to this reference sequence using Bowtie 2.0.4 using the sensitive, end-to-end options.

### SOLiD 4 Sequencing of Blood and Saliva DNA Samples

50 base pair reads were acquired from blood libraries and saliva libraries. SOLiD csfasta and qual files were combined using the solid_to_fastq.py script from Galaxy [45]. The resultant fastq files were mapped to hg19, the PhAnToMe PhageSeed database (http://phantome.org/downloaded on 09/01/2012) and the Human Oral Microbiome Database (HOMD) [20] using Bowtie 0.12.7 with parameters allowing 2 mismatches in a 28 bp seed region. The MetaPhlAn [21] database was manually indexed using Bowtie 0.12.7 tools to allow alignment of SOLiD colorspace reads.

### Illumina Sequencing of Black Molly DNA Sample

Fastq reads from a 66 bp MiSeq run and a 50 bp GAIIx run were combined and analyzed with MG-RAST [23]. Relative abundance was calculated by number of hits on each bacterial genus reported by the total number of bacterial hits. For the MetaPhlAn analysis, reads were trimmed using Sickle (available at https://github.com/vsbuffalo/sickle) until quality scores from 50 bases averaged at least Q20 (Sanger units). These reads were mapped to the MetaPhlAn database using the Bowtie 2 [16] “very sensitive” parameter set.

### Shannon-Weiner Diversity Index Calculation

The Shannon-Weiner Diversity, H′, was calculated using the following equation:

H′ = −**Σ** pi ln(pi) where pi is the proportion of each species in the sample. 149 species were used in the calculation from the saliva HOMD dataset.

## Supporting Information

Figure S1 Analysis of SOLiD 4 reads from blood samples mapping to human chromosomes before and after enrichment with MBD-Fc protein A paramagnetic beads shows a large increase of mitochondrial reads in the enriched dataset. (A) Graph of the fraction of total reads mapped to each chromosome from the unenriched and enriched samples showing a 124-fold increase in reads mapping to mitochondria in the enriched sample. (B) Table displaying total number of reads and the ratio of total reads to mitochondrial reads in unenriched and enriched samples.
(TIF)

Table S1 List of bacterial species reported by MetaPhlAn for saliva experiment.
(CSV)

Table S2 List of bacterial species reported by MetaPhlAn for blood experiment.
(CSV)

Table S3 List of viral species identified by aligning saliva reads to the PhageSeed database.
(CSV)

Table S4 List of bacterial species reported by MG-RAST and MetaPhlAn for black molly experiment.
(CSV)

## References

[1] Bäckhed F, Ley RE, Sonnenburg JL, Peterson DA, Gordon JI (2005) Host-bacterial mutualism in the human intestine. Science (New York, NY) 307: 1915–1920. Available: http://www.ncbi.nlm.nih.gov/pubmed/15790844. Accessed 23 May 2013. 10.1126/science.1104816 15790844

[2] Fujimura KE, Slusher NA, Cabana MD, Lynch SV (2010) Role of the gut microbiota in defining human health. Expert Review of Anti-infective Therapy 8: 435–454. Available: http://www.pubmedcentral.nih.gov/articlerender.fcgi?artid=2881665&tool=pmcentrez&rendertype=abstract. Accessed 20 June 2013. 10.1586/eri.10.14 PMC2881665 20377338

[3] Koren O, Spor A, Felin J, Fåk F, Stombaugh J, et al. (2011) Human oral, gut, and plaque microbiota in patients with atherosclerosis. Proceedings of the National Academy of Sciences of the United States of America 108 Suppl: 4592–4598. Available: http://www.pubmedcentral.nih.gov/articlerender.fcgi?artid=3063583&tool=pmcentrez&rendertype=abstract. Accessed 26 May 2013. 10.1073/pnas.1011383107 PMC3063583 20937873

[4] Liu B, Faller LL, Klitgord N, Mazumdar V, Ghodsi M, et al. (2012) Deep sequencing of the oral microbiome reveals signatures of periodontal disease. PloS One 7: e37919. Available: http://www.pubmedcentral.nih.gov/articlerender.fcgi?artid=3366996&tool=pmcentrez&rendertype=abstract. Accessed 27 May 2013. 10.1371/journal.pone.0037919 PMC3366996 22675498

[5] Kinross JM, Darzi AW, Nicholson JK (2011) Gut microbiome-host interactions in health and disease. Genome Medicine 3: 14. Available: http://www.pubmedcentral.nih.gov/articlerender.fcgi?artid=3092099&tool=pmcentrez&rendertype=abstract. Accessed 16 July 2013. 10.1186/gm228 PMC3092099 21392406

[6] Peterson J, Garges S, Giovanni M, McInnes P, Wang L, et al. (2009) The NIH Human Microbiome Project. Genome Research 19: 2317–2323. Available: http://www.pubmedcentral.nih.gov/articlerender.fcgi?artid=2792171&tool=pmcentrez&rendertype=abstract. Accessed 24 May 2013. 10.1101/gr.096651.109 PMC2792171 19819907

[7] Oliver JD (2010) Recent findings on the viable but nonculturable state in pathogenic bacteria. FEMS Microbiology Reviews 34: 415–425. Available: http://www.ncbi.nlm.nih.gov/pubmed/20059548. Accessed 28 May 2013. 10.1111/j.1574-6976.2009.00200.x 20059548

[8] Shah N, Tang H, Doak TG, Ye Y (2011) Comparing bacterial communities inferred from 16S rRNA gene sequencing and shotgun metagenomics. Pacific Symposium on Biocomputing Pacific Symposium on Biocomputing: 165–176. Available: http://www.ncbi.nlm.nih.gov/pubmed/21121044. Accessed 25 June 2013. 10.1142/9789814335058_0018 21121044

[9] Diaz PI, Dupuy AK, Abusleme L, Reese B, Obergfell C, et al. (2012) Using high throughput sequencing to explore the biodiversity in oral bacterial communities. Molecular Oral Microbiology 27: 182–201. Available: http://www.ncbi.nlm.nih.gov/pubmed/22520388. Accessed 21 May 2013. 10.1111/j.2041-1014.2012.00642.x PMC3789374 22520388

[10] Lewis JD, Meehan RR, Henzel WJ, Maurer-Fogy I, Jeppesen P, et al. (1992) Purification, sequence, and cellular localization of a novel chromosomal protein that binds to methylated DNA. Cell 69: 905–914. Available: http://www.ncbi.nlm.nih.gov/pubmed/1606614. Accessed 26 June 2013. 10.1016/0092-8674(92)90610-o 1606614

[11] Hendrich B, Bird A (1998) Identification and characterization of a family of mammalian methyl-CpG binding proteins. Molecular and Cellular Biology 18: 6538–6547. Available: http://www.pubmedcentral.nih.gov/articlerender.fcgi?artid=109239&tool=pmcentrez&rendertype=abstract. Accessed 26 June 2013. 10.1128/mcb.18.11.6538 PMC109239 9774669

[12] Gebhard C, Schwarzfischer L, Pham T-H, Schilling E, Klug M, et al. (2006) Genome-wide profiling of CpG methylation identifies novel targets of aberrant hypermethylation in myeloid leukemia. Cancer Research 66: 6118–6128. Available: http://www.ncbi.nlm.nih.gov/pubmed/16778185. Accessed 5 June 2013. 10.1158/0008-5472.CAN-06-0376 16778185

[13] Estève P-O, Chin HG, Benner J, Feehery GR, Samaranayake M, et al (2009) Regulation of DNMT1 stability through SET7-mediated lysine methylation in mammalian cells. Proceedings of the National Academy of Sciences of the United States of America 106: 5076–5081 Available: http://www.pubmedcentral.nih.gov/articlerender.fcgi?artid=2654809&tool=pmcentrez&rendertype=abstract. 19282482 10.1073/pnas.0810362106 PMC2654809

[14] International Human Genome Sequencing Consortium (2004) Finishing the euchromatic sequence of the human genome. Nature 431: 931–945. Available: 10.1038/nature03001. Accessed 21 May 2013. 15496913

[15] Riley M, Abe T, Arnaud MB, Berlyn MKB, Blattner FR, et al. (2006) Escherichia coli K-12: a cooperatively developed annotation snapshot–2005. Nucleic Acids Research 34: 1–9. Available: http://www.pubmedcentral.nih.gov/articlerender.fcgi?artid=1325200&tool=pmcentrez&rendertype=abstract. Accessed 29 May 2013. 10.1093/nar/gkj405 PMC1325200 16397293

[16] Langmead B, Salzberg SL (2012) Fast gapped-read alignment with Bowtie 2. Nature Methods 9: 357–359. Available: http://www.pubmedcentral.nih.gov/articlerender.fcgi?artid=3322381&tool=pmcentrez&rendertype=abstract. Accessed 22 May 2013. 10.1038/nmeth.1923 PMC3322381 22388286

[17] Lister R, Pelizzola M, Dowen RH, Hawkins RD, Hon G, et al (2009) NIH Public Access. Nature 462: 315–322 10.1038/nature08514.Human 19829295 PMC2857523

[18] Fang G, Munera D, Friedman DI, Mandlik A, Chao MC, et al. (2012) Genome-wide mapping of methylated adenine residues in pathogenic Escherichia coli using single-molecule real-time sequencing. Nature Biotechnology 30: 1232–1239. Available: http://www.ncbi.nlm.nih.gov/pubmed/23138224. Accessed 23 May 2013. 10.1038/nbt.2432 PMC3879109 23138224

[19] Murray IA, Clark TA, Morgan RD, Boitano M, Anton BP, et al. (2012) The methylomes of six bacteria. Nucleic Acids Research 40: 11450–11462. Available: http://www.pubmedcentral.nih.gov/articlerender.fcgi?artid=3526280&tool=pmcentrez&rendertype=abstract. Accessed 22 May 2013. 10.1093/nar/gks891 PMC3526280 23034806

[20] Chen T, Yu W-H, Izard J, Baranova O V, Lakshmanan A, et al. (2010) The Human Oral Microbiome Database: a web accessible resource for investigating oral microbe taxonomic and genomic information. Database: the Journal of Biological Databases and Curation 2010: baq013. Available: http://www.pubmedcentral.nih.gov/articlerender.fcgi?artid=2911848&tool=pmcentrez&rendertype=abstract. Accessed 21 May 2013. 10.1093/database/baq013 PMC2911848 20624719

[21] Segata N, Waldron L, Ballarini A, Narasimhan V, Jousson O, et al (2012) Metagenomic microbial community profiling using unique clade- specific marker genes. Nature Methods 9: 811–813 10.1038/Nmeth.2066 22688413 PMC3443552

[22] Ritchot N, Roy PH (1990) DNA methylation in Neisseria gonorrhoeae and other Neisseriae. Gene 86: 103–106. Available: 10.1016/0378-1119(90)90120-G. Accessed 11 July 2013. 2155857

[23] Meyer F, Paarmann D, D’Souza M, Olson R, Glass EM, et al. (2008) The metagenomics RAST server - a public resource for the automatic phylogenetic and functional analysis of metagenomes. BMC Bioinformatics 9: 386. Available: http://www.pubmedcentral.nih.gov/articlerender.fcgi?artid=2563014&tool=pmcentrez&rendertype=abstract. Accessed 24 May 2013. 10.1186/1471-2105-9-386 PMC2563014 18803844

[24] Gnerre S, Maccallum I, Przybylski D, Ribeiro FJ, Burton JN, et al. (2011) High-quality draft assemblies of mammalian genomes from massively parallel sequence data. Proceedings of the National Academy of Sciences of the United States of America 108: 1513–1518. Available: http://www.pubmedcentral.nih.gov/articlerender.fcgi?artid=3029755&tool=pmcentrez&rendertype=abstract. Accessed 22 May 2013. 10.1073/pnas.1017351108 PMC3029755 21187386

[25] Verner-Jeffreys DW, Welch TJ, Schwarz T, Pond MJ, Woodward MJ, et al. (2009) High prevalence of multidrug-tolerant bacteria and associated antimicrobial resistance genes isolated from ornamental fish and their carriage water. PloS One 4: e8388. Available: http://www.pubmedcentral.nih.gov/articlerender.fcgi?artid=2793012&tool=pmcentrez&rendertype=abstract. Accessed 23 May 2013. 10.1371/journal.pone.0008388 PMC2793012 20027306

[26] Weir M, Rajić A, Dutil L, Cernicchiaro N, Uhland FC, et al. (2012) Zoonotic bacteria, antimicrobial use and antimicrobial resistance in ornamental fish: a systematic review of the existing research and survey of aquaculture-allied professionals. Epidemiology and Infection 140: 192–206. Available: http://www.ncbi.nlm.nih.gov/pubmed/21906415. Accessed 15 July 2013. 10.1017/S0950268811001798 21906415

[27] Austin B, Austin DDA (2007) Bacterial Fish Pathogens: Diseases of Farmed and Wild Fish (Google eBook). Springer. Available: http://books.google.com/books?hl=en&lr=&id=gOdFZqogvMQC&pgis=1. Accessed 15 July 2013.

[28] Dawid IB (1974) 5-methylcytidylic acid: absence from mitochondrial DNA of frogs and HeLa cells. Science (New York, NY) 184: 80–81. Available: http://www.ncbi.nlm.nih.gov/pubmed/4815287. Accessed 27 June 2013. 10.1126/science.184.4132.80 4815287

[29] Shmookler Reis RJ, Goldstein S (1983) Mitochondrial DNA in mortal and immortal human cells. Genome number, integrity, and methylation. The Journal of Biological Chemistry 258: 9078–9085. Available: http://www.ncbi.nlm.nih.gov/pubmed/6307991. Accessed 27 June 2013. 6307991

[30] Horz H-P, Scheer S, Vianna ME, Conrads G (2010) New methods for selective isolation of bacterial DNA from human clinical specimens. Anaerobe 16: 47–53. 19463963 10.1016/j.anaerobe.2009.04.009

[31] Bright AT, Tewhey R, Abeles S, Chuquiyauri R, Llanos-Cuentas A, et al. (2012) Whole genome sequencing analysis of Plasmodium vivax using whole genome capture. BMC Genomics 13: 262. Available: http://www.pubmedcentral.nih.gov/articlerender.fcgi?artid=3410760&tool=pmcentrez&rendertype=abstract. Accessed 6 June 2013. 10.1186/1471-2164-13-262 PMC3410760 22721170

[32] Auburn S, Campino S, Clark TG, Djimde AA, Zongo I, et al. (2011) An effective method to purify Plasmodium falciparum DNA directly from clinical blood samples for whole genome high-throughput sequencing. PloS One 6: e22213. Available: http://www.pubmedcentral.nih.gov/articlerender.fcgi?artid=3138765&tool=pmcentrez&rendertype=abstract. Accessed 17 July 2013. 10.1371/journal.pone.0022213 PMC3138765 21789235

[33] Oyola SO, Gu Y, Manske M, Otto TD, O’Brien J, et al. (2013) Efficient depletion of host DNA contamination in malaria clinical sequencing. Journal of Clinical Microbiology 51: 745–751. Available: http://www.pubmedcentral.nih.gov/articlerender.fcgi?artid=3592063&tool=pmcentrez&rendertype=abstract. Accessed 22 May 2013. 10.1128/JCM.02507-12 PMC3592063 23224084

[34] Tringe SG, Hugenholtz P (2008) A renaissance for the pioneering 16S rRNA gene. Current Opinion in Microbiology 11: 442–446. Available: http://www.ncbi.nlm.nih.gov/pubmed/18817891. Accessed 22 May 2013. 10.1016/j.mib.2008.09.011 18817891

[35] Morgan XC, Segata N, Huttenhower C (2013) Biodiversity and functional genomics in the human microbiome. Trends in Genetics: TIG 29: 51–58. Available: http://www.ncbi.nlm.nih.gov/pubmed/23140990. Accessed 25 May 2013. 10.1016/j.tig.2012.09.005 PMC3534939 23140990

[36] Camacho C, Coulouris G, Avagyan V, Ma N, Papadopoulos J, et al. (2009) BLAST+: architecture and applications. BMC Bioinformatics 10: 421. Available: http://www.pubmedcentral.nih.gov/articlerender.fcgi?artid=2803857&tool=pmcentrez&rendertype=abstract. Accessed 3 June 2013. 10.1186/1471-2105-10-421 PMC2803857 20003500

[37] Lazarevic V, Whiteson K, Gaïa N, Gizard Y, Hernandez D, et al. (2012) Analysis of the salivary microbiome using culture-independent techniques. Journal of Clinical Bioinformatics 2: 4. Available: http://www.pubmedcentral.nih.gov/articlerender.fcgi?artid=3296672&tool=pmcentrez&rendertype=abstract. Accessed 20 June 2013. 10.1186/2043-9113-2-4 PMC3296672 22300522

[38] Human Microbiome Project Consortium (2012) A framework for human microbiome research. Nature 486: 215–221. Available: http://www.pubmedcentral.nih.gov/articlerender.fcgi?artid=3377744&tool=pmcentrez&rendertype=abstract. Accessed 7 June 2013. 10.1038/nature11209 PMC3377744 22699610

[39] Boudraa M, Perrin P (1987) CpG and TpA frequencies in the plant system. Nucleic Acids Research 15: 5729–5737. Available: http://www.pubmedcentral.nih.gov/articlerender.fcgi?artid=306018&tool=pmcentrez&rendertype=abstract. Accessed 27 June 2013. 10.1093/nar/15.14.5729 PMC306018 3497385

[40] Hong EE, Okitsu CY, Smith AD, Hsieh C-L (2013) Regionally-Specific and Genome-Wide Analyses Conclusively Demonstrate the Absence of CpG Methylation In Human Mitochondrial DNA. Molecular and Cellular Biology 33: 2683–2690. Available: http://www.ncbi.nlm.nih.gov/pubmed/23671186. Accessed 23 May 2013. 10.1128/MCB.00220-13 PMC3700126 23671186

[41] Shock LS, Thakkar P V, Peterson EJ, Moran RG, Taylor SM (2011) DNA methyltransferase 1, cytosine methylation, and cytosine hydroxymethylation in mammalian mitochondria. Proceedings of the National Academy of Sciences of the United States of America 108: 3630–3635. Available: http://www.pubmedcentral.nih.gov/articlerender.fcgi?artid=3048134&tool=pmcentrez&rendertype=abstract. Accessed 21 May 2013. 10.1073/pnas.1012311108 PMC3048134 21321201

[42] Cardon LR, Burge C, Clayton DA, Karlin S (1994) Pervasive CpG suppression in animal mitochondrial genomes. Proceedings of the National Academy of Sciences of the United States of America 91: 3799–3803. Available: http://www.pubmedcentral.nih.gov/articlerender.fcgi?artid=43669&tool=pmcentrez&rendertype=abstract. Accessed 27 June 2013. 10.1073/pnas.91.9.3799 PMC43669 8170990

[43] Chaves A, Vergara C, Mayer J (1995) Dichloromethane as an economic alternative to chloroform in the extraction of DNA from plant tissues. Plant Molecular Biology Reporter 13: 18–25 Available: 10.1007/BF02668389.

[44] Oyola SO, Otto TD, Gu Y, Maslen G, Manske M, et al. (2012) Optimizing Illumina next-generation sequencing library preparation for extremely AT-biased genomes. BMC Genomics 13: 1. Available: http://www.pubmedcentral.nih.gov/articlerender.fcgi?artid=3312816&tool=pmcentrez&rendertype=abstract. Accessed 23 May 2013. 10.1186/1471-2164-13-1 PMC3312816 22214261

[45] Goecks J, Eberhard C, Too T, Nekrutenko A, Taylor J (2013) Web-based visual analysis for high-throughput genomics. BMC Genomics 14: 397. Available: http://www.pubmedcentral.nih.gov/articlerender.fcgi?artid=3691752&tool=pmcentrez&rendertype=abstract. Accessed 13 June 2013. 10.1186/1471-2164-14-397 PMC3691752 23758618

[46] Nikolskaya II, Tkatcheva ZG, Vanyushin BF, Tikchonenko TI (1968) On the presence of minor bases in DNA of phages DD7 and Sd and their hosts. Biochimica et Biophysica Acta 155: 626–629. Available: http://www.ncbi.nlm.nih.gov/pubmed/4866308. Accessed 27 June 2013. 10.1016/0005-2787(68)90211-6 4866308

