# Supplementary figures and images for: A Method for Selectively Enriching Microbial DNA from Contaminating Vertebrate Host DNA
Source: PLoS One. 2013 Oct 28;8(10):e76096. doi: 10.1371/journal.pone.0076096 (PMC3810253; doi:10.1371/journal.pone.0076096)

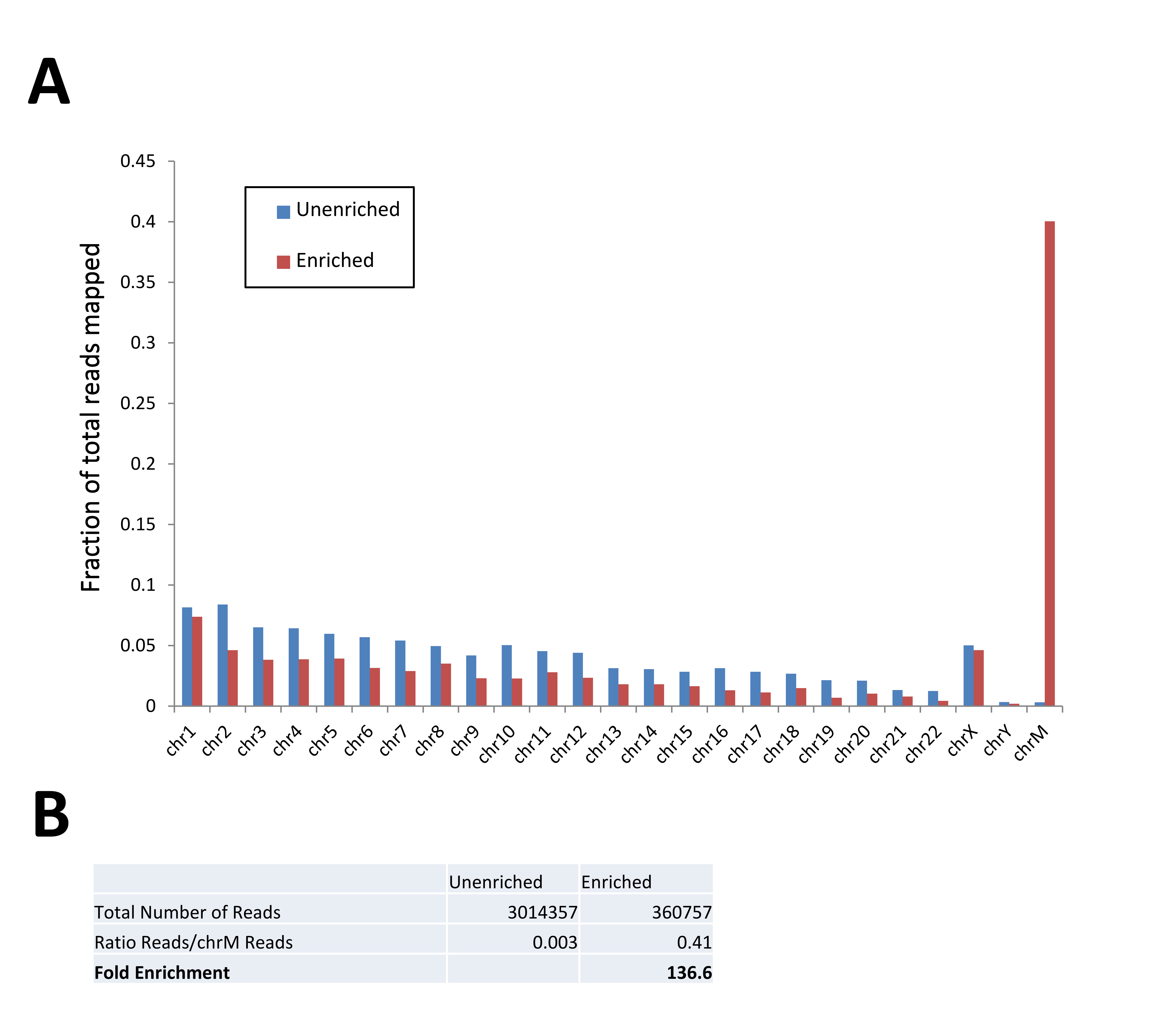

Supplement: Figure S1 — Analysis of SOLiD 4 reads from blood samples mapping to human chromosomes before and after enrichment with MBD-Fc protein A paramagnetic beads shows a large increase of mitochondrial reads in the enriched dataset. (A) Graph of the fraction of total reads mapped to each chromosome from the unenriched and enriched samples showing a 124-fold increase in reads mapping to mitochondria in the enriched sample. (B) Table displaying total number of reads and the ratio of total reads to mitochondrial reads in unenriched and enriched samples. (TIF) [file pone.0076096.s001.tif]
